# Posttranslational modifications of α-tubulin in alzheimer disease

**DOI:** 10.1186/s40035-015-0030-4

**Published:** 2015-05-15

**Authors:** Fan Zhang, Bo Su, Chunyu Wang, Sandra L. Siedlak, Siddhartha Mondragon-Rodriguez, Hyoung-gon Lee, Xinglong Wang, George Perry, Xiongwei Zhu

**Affiliations:** Department of Pathology, Case Western Reserve University, Cleveland, OH 44121 USA; Department of Neurosurgery, Shandong Provincial Hospital, Shandong University, Jinan, 250012 China; Department of Neurobiology, Shandong University, Jinan, 250012 China; Department of Neurology, the Second Xiangya Hospital, Central South University, Changsha, Hunan 410011 China; Departamento de Neurobiología del Desarrollo y Neurofisiología, Instituto de Neurobiología, Universidad Nacional Autónoma de México Querétaro, Querétaro México, D. F. Mexico; The University of Texas at San Antonio, One UTSA Circle, San Antonio, TX 78249 USA; 2103 Cornell Road, Cleveland, OH 44106 USA

**Keywords:** Acetylation, Alzheimer disease, Polyglutamylation, Tau, Tubulin

## Abstract

**Background:**

In Alzheimer disease (AD), hyperphosphorylation of tau proteins results in microtubule destabilization and cytoskeletal abnormalities. Our prior ultra-morphometric studies documented a clear reduction in microtubules in pyramidal neurons in AD compared to controls, however, this reduction did not coincide with the presence of paired helical filaments. The latter suggests the presence of compensatory mechanism(s) that stabilize microtubule dynamics despite the loss of tau binding and stabilization. Microtubules are composed of tubulin dimers which are subject to posttranslational modifications that affect the stability and function of microtubules.

**Methods:**

In this study, we performed a detailed analysis on changes in the posttranslational modifications in tubulin in postmortem human brain tissues from AD patients and age-matched controls by immunoblot and immunocytochemistry.

**Results:**

Consistent with our previous study, we found decreased levels of α-tubulin in AD brain. Levels of tubulin with various posttranslational modifications such as polyglutamylation, tyrosination, and detyrosination were also proportionally reduced in AD brain, but, interestingly, there was an increase in the proportion of the acetylated α-tubulin in the remaining α-tubulin. Tubulin distribution was changed from predominantly in the processes to be more accumulated in the cell body. The number of processes containing polyglutamylated tubulin was well preserved in AD neurons. While there was a cell autonomous detrimental effect of NFTs on tubulin, this is likely a gradual and slow process, and there was no selective loss of acetylated or polyglutamylated tubulin in NFT-bearing neurons.

**Conclusions:**

Overall, we suggest that the specific changes in tubulin modification in AD brain likely represent a compensatory response.

## Background

Alzheimer disease (AD), as the most common neurodegenerative disease, is characterized by the pathological markers such as intracellular neurofibrillary tangles (NFTs) and extracellular senile plaques. NFTs are mainly composed of a highly phosphorylated form of the microtubule associated protein tau, and senile plaques are primarily composed of amyloid-β. Physiologically, tau regulates microtubule stability by binding to microtubules. Phosphorylation and dephosphorylation of tau at specific sites such as Ser262 or Thr231 regulates its binding ability to microtubules [1, 2]. In AD patients, hyperphosphorylated tau proteins have low tubulin-binding activity and form paired helical filaments which are believed to lead to microtubule destabilization and cytoskeletal abnormalities [3].

Our previous ultra-morphometric study demonstrated that microtubules are significantly reduced in number and length in AD neurons, however, their loss does not correspond with the formation of paired helical filaments [4]. In fact, abundant microtubules were often seen in close juxtaposition to paired helical filaments, suggesting that microtubule deficit is independent of tau filament formation [4]. Even though the overall function of microtubules and cellular actions dependent on microtubules including axonal transport are likely compromised [5–8], neurons continue to be functionally integrated and survive despite increased levels of phosphorylated tau proteins and deposited filaments [9, 10]. This suggests the presence of mechanism(s) compensating for the loss of tau binding/stabilizing activity affecting microtubules in these neurons.

Microtubules are composed of tubulin heterodimers made of α- and β-tubulin. The C-terminal tail of α-tubulin is subject to posttranslational modifications such as detyrosination, acetylation, and polyglutamylation, which affects the function and stability of microtubules [11, 12]. We hypothesize that compensatory changes in posttranslational modification of tubulin could alleviate deficits induced by microtubule destabilization/reduction in susceptible neurons in AD brain. To begin to test this hypothesis, we performed a detailed immunoblot and immunocytochemical analysis to investigate various posttranslational modifications to tubulin in the brain tissue from AD and control patients.

## Methods

### Human tissues and Immunocytochemistry

Human brain tissue samples were obtained postmortem from patients with histopathologically-confirmed AD (n = 3) (see Table 1) and non-AD controls (n = 4. Except for the lack of NFTs, the young control case (C1) demonstrated similar staining pattern as other controls cases for all the antibodies used). Tissue was fixed in methacarn (methanol:chloroform:acetic acid in a 6:3:1 ratio) immersion for 24 h at 4 °C. Tissue was subsequently dehydrated through graded ethanol and xylene solutions, embedded in paraffin, and sectioned at 6 μm. Following hydration, sequential sections were immunostained by the peroxidase-antiperoxidase procedure with DAB as chromogen [13] using mouse monoclonal antibodies against α-tubulin (Epitomics, Burlingame, CA, USA), acetylated tubulin (Sigma, St. Louis, MO, USA, product#T6793), tyrosinated tubulin (Sigma, Product# T9028), detyrosinated tubulin (Chemicon, cata#MAB5566) and polyglutamylated tubulin (Sigma, Product#T9822). Sections were also double stained for NFT using a rabbit antibody against tau protein and the alkaline phosphatase anti alkaline phosphatase method and developed with Fast Blue.

**Table 1:** Details of Alzheimer disease and control cases used in the immunocytochemical studies

Case	Neuropathological Diagnosis	Gender	Age	# NFT/mm^2^ CA1
AD 1	Alzheimer disease	F	76	55.4
AD 2	Alzheimer disease, severe	F	77	81.6
AD 3	Alzheimer disease	F	88	29.9
C 1	No pathological diagnosis	M	62	0
C 2	Infarcts	F	69	0.8
C 3	No pathological diagnosis	F	74	8.1
C 4	No pathological diagnosis	M	81	7.8

### Double-label immunofluorescence images

Following rehydration brain tissue sections were blocked with 10 % normal goat serum in phosphate-buffered saline for 1 h, then incubated with primary antibody pSer396 (Biosource, Camarillo, CA, USA. 1:200) and acetylated tubulin overnight at 4 °C. Following three washes, the sections were incubated with 488/564-conjugated secondary antibody (Invitrogen, Grand Island, NY, USA) (1:500) for 1 h at 37 °C in the dark. Tissues were rinsed three times with phosphate-buffered saline and mounted with antifade medium (Southern Biotech, Birmingham, AL, USA). All fluorescence images were captured with a Zeiss LSM 510 inverted fluorescence microscope or a Zeiss LSM 510 inverted laser-scanning confocal fluorescence microscope.

### Western blotting

Samples of frozen gray matter of hippocampus of AD (n = 9, age 78.3 ± 1.7, postmortem interval of 6 ± 1.6 h) and control cases (n = 8, ages 74.1 ± 4.7, postmortem interval of 7.4 ± 2 h, there was no significant difference in the age and postmortem intervals between AD and control groups) were homogenized in 10 x volumes of lysis buffer (Cell Signaling, Danvers, MA, USA) and centrifuged for 10 min at 16,000 x g. Protein concentration of the supernatants was determined by the bicinchoninic acid assay method (Pierce, Rockford, IL, USA). Western blot was performed to examine α-tubulin, acetylated tubulin, tyrosine tubulin, polyglutamylated tubulin, detyrosinated tubulin, and glyceraldehyde 3-phosphate dehydrogenase (GAPDH) (Millipore, Bedford, MA, USA) levels in samples.

Blots were scanned at high resolution and the immunoreactive bands were quantitated with Quantity One software (Bio-rad, Hercules, CA, USA). The quantification results (means ± SEM) were analyzed used the Student's *t*-test to determine the significance (*p* < 0.05).

## Results

**Fig. 1:**
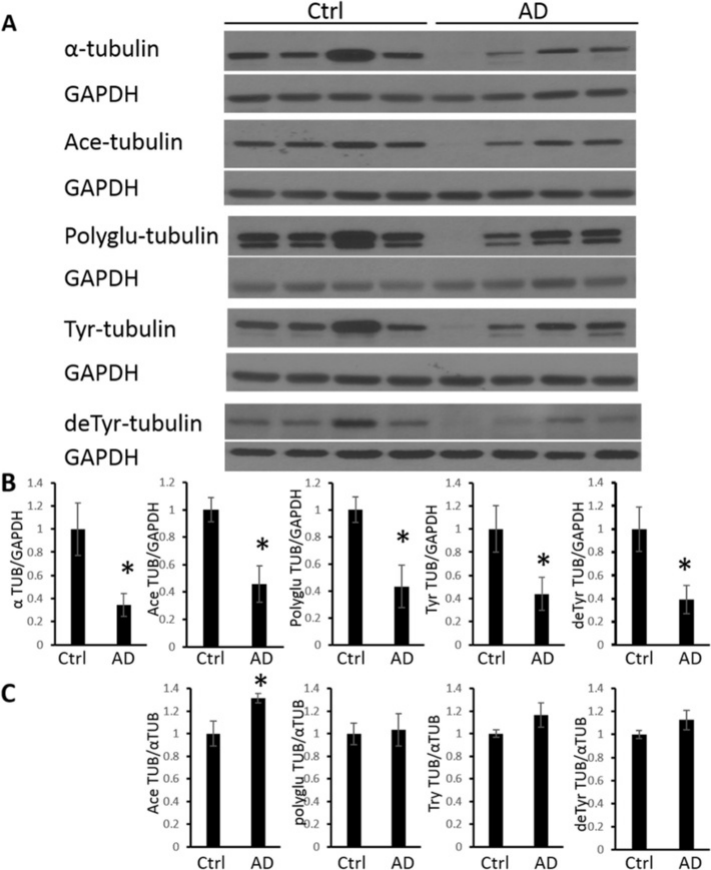
Immunoblot analysis of tubulins in AD brain. (**a**) Representative immunoblot analysis of tubulin expression and post-translational modifications in brain homogenates from hippocampal tissues from AD and age-matched control patients. GAPDH was used as the internal loading control. (**b**) The quantification results, normalized to GAPDH levels, confirmed a significant decrease in α-tubulin, acetylated tubulin, polyGlu-tubulin, tyrosinated tubulin, and detyr-tubulin levels. (**c**) The quantification results, normalized to α-tubulin levels, demonstrated an increase in acetylated tubulin (Ace TUB). Data are means ± SEM. * indicates significant difference between AD and control with *p* < 0.05

Levels of post-translational modifications of tubulin in the hippocampus, including acetylated tubulin, tyrosinated tubulin, detyrosinated tubulin, and polyglutamylated tubulin along with total expression levels of α-tubulin, were determined by western blot (Fig. 1a). Expression levels of GAPDH were also determined by western blot as an internal loading control. Quantitative analysis, normalized to the levels of GAPDH (Fig. 1b), revealed that levels of total α-tubulin were significantly reduced by approximately 65 % in the brains from AD patients compared to age-matched control brains. Similarly, levels of acetylated tubulin, polyglutamylated tubulin, tyrosinated tubulin, and detyrosinated tubulin were also significantly reduced in AD brain (Fig. 1b). The significant reduction of tyrosinated tubulin, detryosinated tubulin, and polygluatmylated tubulin in the AD brains was proportional to the reduction of the total α-tubulin since there was no difference between AD and control brains when the levels of these modified tubulins were normalized to total α-tubulin (Fig. 1c). However, when normalized to total α-tubulin, the ratio of acetylated tubulin was significantly increased by approximately 31 % in AD compared to controls (Fig. 1c), suggesting that acetylated tubulin is more resistant to degradation in AD.

**Fig. 2:**
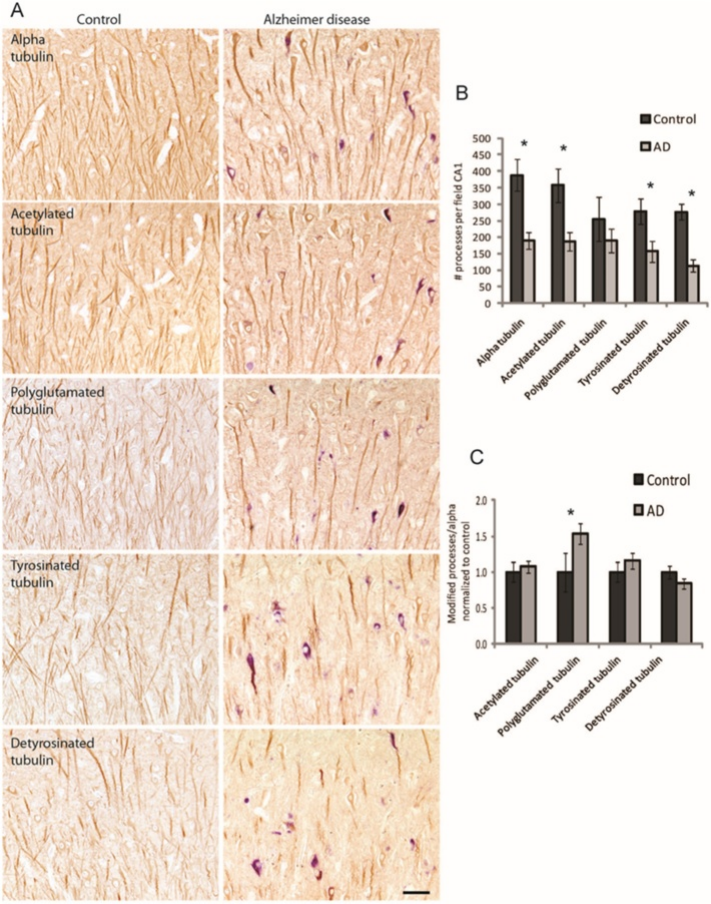
Immunocytochemical analysis of tubulins in AD brain. Representative images of the CA1 region demonstrate specific staining of the tubulin antibodies for neuron cell bodies and axonal processes in both control and AD cases (**a**). Tubulin antibodies are stained brown and NFT, using tau antibody, are stained blue. Qualitatively it appears that there are fewer processes stained in the AD cases and that only the thicker processes are retained (A). Quantification found there are significantly fewer processes stained for alpha, acetylated, tyrosinated, and detyrosinated tubulin in AD cases (**b**). When normalized to α-tubulin levels, an increase in stable glutamylated tubulin was found in AD cases (**c**). *p < 0.05. Scale bar = 50 μm

We next examined the localization of the various tubulin populations in AD and control hippocampal sections by immunocytochemistry. At the light level, all cases examined showed clear and specific immunostaining for each of the monoclonal tubulin modification antibodies. The same region of the CA1 was shown for each antibody in a control case with no NFT and in an AD case with blue-stained NFT (Fig. 2a). All the tubulin antibodies stained many long axonal processes plus finer processes between axons and occasional neuronal cell bodies in the control cases. The AD cases appeared to have fewer axons stained but the cell bodies were more apparent. Further, qualitatively, there appeared to be fewer of the finer processes immunostained in the AD cases, such that only the thicker processes were stained.

Quantification of the immunostained axonal processes in the CA1 region by each of these tubulin antibodies found there was less tubulin in the AD cases. The number of processes stained was significantly lower in the CA1 for the α-tubulin, and the acetylated, tyrosinated, and detyrosinated modifications while there was only a trend of reduction for the polyglutamylated tubulin that did not reach significance (Fig. 2b). Within each case, it was possible to directly compare how each modification was maintained in the CA1 neuronal population relative to α-tubulin. No significant difference was found in the proportion of processes with acetylated, tyrosinated, or detyrosinated tubulin between AD and control, but the proportion of processes with polyglutamylated tubulin was significantly increased in AD (Fig. 2c).

**Fig. 3:**
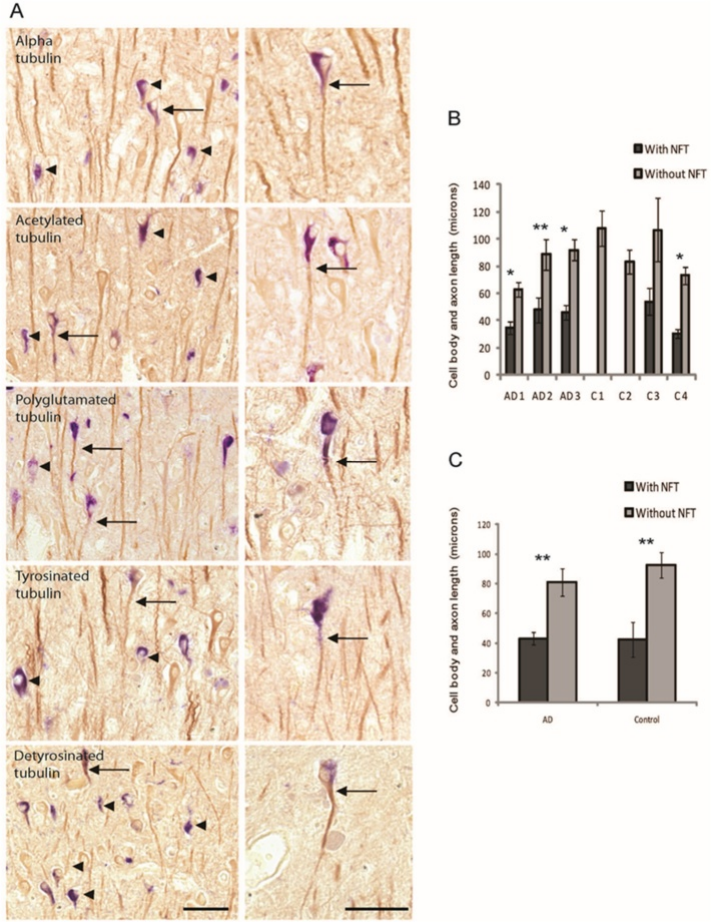
The relationship between neuronal tubulin levels and NFTs. Most neurons with NFT (mean 78 %) did not demonstrate axonal processes stained for tubulins (**a**, arrowheads left panels). However, some neurons with NFT (blue), had tubulin staining in the cell body and axonal process (A, arrows). Every tubulin modification was retained in the axonal process in some NFT-bearing neurons (A, right panels). Scale bars = 50 μm. Measuring the lengths of the cell bodies and axonal processes in the NFT and normal surrounding neurons in the CA1 region found the NFTs were indeed significantly shorter (**b**) in all AD cases and one control case. Taken together, the NFT in both AD and control cases, were significantly shorter when compared to the normal neuron population (**c**), yet no difference in length was found when comparing AD and control NFT, or AD and control normal neurons, suggesting the loss of axonal process length is a result of NFT formation and not disease. *p < 0.001, **p < 0.05

To discern the potential effects of NFTs on tubulin expression and modifications, double staining methods were employed. For each modification, other than the few ghost NFTs, all the NFT-bearing neurons contained various levels of tubulins, either in the axons or also in the cell body. Among all cases examined, the majority of NFT-bearing neurons counted in the entire CA1 region (on average around 78 %) were lacking any axonal process stained for tubulin, yet maintained some tubulin immunoreactivity in the cell body (arrowheads in Fig. 3a, left panels), however, for each modification, there were still some NFT-bearing neurons (on average around 22 %) with long axonal processes stained for tubulins (Fig. 3a, right panels, arrows). The pathological tau (stained blue) was restricted to the cell body and only a short distance down the axon. Measuring the length of the cell body and stained axonal processes revealed significantly shorter neurons/processes when NFT were present, compared to all surrounding tubulin-positive cells lacking NFT in all AD cases and the control cases with NFT (Fig. 3b,c). Yet, no difference was noted in these normal neurons or NFT-bearing neurons between the AD and control cases (Fig. 3c), suggesting the neuronal tubulin morphology changes are a reflection of NFT pathology and not disease state.

**Fig. 4:**
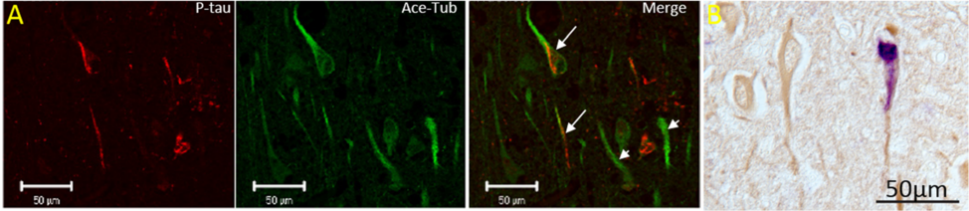
NFT-bearing neurons do not necessarily contain less acetylated tubulin. (**a**) Confocal microscopy demonstrated that in AD hippocampal tissues, those neurons containing neurofibrillary pathology (red, arrowheads) display levels of acetylated tubulin (green) comparable to those without NFT (arrows). (**b**) The same pattern was found using light level microscopy with acetylated tubulin stained brown, and phospho-tau stained blue

**Fig. 5:**
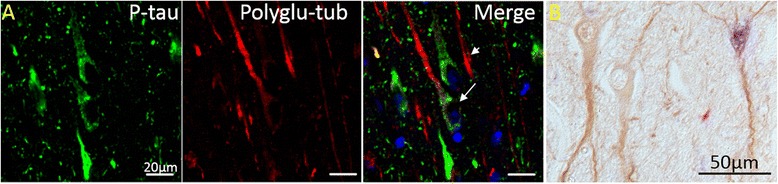
NFT-bearing neurons do not necessarily contain less polyglutamylated tubulin. (**a**) Levels of polyglutamylated tubulin (red) are similar in both normal and NFT-bearing neurons stained with phosphorylated tau (green). Blue: DAPI. (**b**) The same pattern was found using light level microscopy with glutamylated tubulin stained brown, and phospho-tau stained blue

It was previously reported that NFT-bearing neurons contain less acetylated tubulin [14]. However, such a pattern was not confirmed in our study as various levels of acetylated tubulin were observed in NFT-bearing neurons similar to that of the NFT-free neurons. NFT-bearing neurons with comparable levels of acetylated tubulin as compared to those neighboring neurons without NFTs were frequently observed. This staining pattern was noted in using light level immunohistochemistry and was also confirmed with double label fluorescent microscopy (Fig. 4). Similarly, various levels of polyglutamylated tubulin were also observed in NFT-bearing neurons with many NFT-bearing neurons demonstrating comparable levels of polyglutamylated tubulin as compared to neighboring neurons without NFT, again seen using both staining methodologies (Fig. 5).

## Discussion

One of the key features associated with AD is hyperphosphorylation of tau protein which reduces its binding affinity to microtubules, thus resulting in instability and dysfunction of microtubule and related axonal transport [3]. However, despite the fact that tangle bearing neurons lose substantial amounts of structurally normal microtubules [6, 15], prior studies demonstrated that neurons survive decades in the presence of tangles [16]. This suggests that possible compensatory mechanisms may support a sufficiently efficient microtubule network and axonal transport and/or a gradual loss of essential functions of microtubule network. In the current study, we made several interesting observations: 1) there were significantly reduced levels of α-tubulin along with proportional reduction in the absolute levels of polyglutamylated, tyrosinated, and detyrosinated tubulin in the AD brain; 2) despite the significant reduction in the absolute level, acetylated tubulin was proportionally increased in the remaining α-tubulin in the AD brain; 3) α-tubulin and modified tubulins were more accumulated in the cell bodies and thicker processes in AD neurons compared to predominant distribution in both thicker axonal processes and finer branches in neurons in the control brain; 4) the number of processes decorated by polyglutamylated tubulin was not significantly decreased in AD brain. In fact, it was proportionally and significantly increased in AD when normalized with that of α-tubulin; 5) the majority of NFT-bearing neurons lack tubulin-decorated axons, but there were still significant number of NFT-bearing neurons with such long axons; and 6) there was no correlation between the presence of NFTs and the immunoreactivity of acetylated tubulin or polyglutamylated tubulin in the neurons in AD brain.

The finding of decreased total expression levels of α-tubulin and the decreased number of α-tubulin positive axonal processes in the AD cases in the present work is consistent with our previous ultrastructural analysis study, which shows that both number and total length of microtubules were significantly and selectively reduced in pyramidal neurons from AD in comparison to control cases [4]. Indeed, other deficiencies related to abnormal microtubules such as deficits in fast axonal transport, dystrophic neurites, and abnormal mitochondrial distribution [6, 17–20] are also reported in AD brains, suggesting that decreased α-tubulin expression could contribute to such deficits and to the pathogenesis of AD. It is not clear what the functional significance of increased levels of α-tubulin in the cell body and the proximal end of the axon processes, but it explains the observation of close juxtaposition of abundant microtubules to paired helical filaments [4] since NFTs are normally accumulated in these regions.

One interesting finding in our study is that despite the reduction in the absolute levels of acetylated tubulin in AD brain, when normalized to reduced levels of α-tubulin, there is increased proportion of acetylated tubulin in the remaining α-tubulin in AD. Acetylation occurs after microtubule assembly at the ε-amine of lysine 40 localized on the inside of the microtubule polymers, which is preserved in α-tubulin but not β-tubulin [21, 22]. Acetylated α-tubulin is present in stable, long-lived microtubules with slow dynamics [23]. One interpretation is that microtubules containing acetylated α-tubulin are better preserved than other microtubules in AD brains. This may be due to its distinct localization in mature neurons as it is enriched in the proximal site of the axon and dendrites [24]. Indeed, our results indicated that the thicker axonal processes are better preserved while those finer processes likely representing branches are lost in AD neurons.

The function of tubulin acetylation remains to be fully understood. Although the early studies indicated that acetylation itself does not confer stability unto microtubules, it was difficult to distinguish whether the acetylation dictated microtubule stability or whether stabilized microtubules became more extensively modified [25]. Nevertheless, tubulin acetylation helps in stability by promoting salt bridge formation between adjacent protofilaments [26]. In the presence of tau protein, acetylated tubulin makes microtubule resistant to the action of severing protein katanin [27]. Functional studies demonstrated that acetylation of α-tubulin is essential for the association of motor proteins (i.e., dynein and kinesin) with microtubules and enhances kinesin-based transport in cells [28–30]. However, these observations were not confirmed in purified cell free system [31, 32], suggesting that tubulin acetylation may indirectly impact intracellular transport requiring additional factors in cells. We suspect that the increased proportion of acetylated tubulin in AD may represent an adaptive change in compensation for the loss of microtubules and their associated deficits in axonal transport along microtubules. Such a notion is supported by the finding that acetylated tubulin can be stress-induced in the hippocampus [33] and tubulin hyperacetylation appears to be a common response to several cellular stresses by modulating the binding and function of signaling factors essential for cell survival [34–36]. In this regard, it is of interest to note that inhibition of histone deacetylase 6 (HDAC6), the major tubulin deacetylase, increased the amount of acetylated tubulin and concomitantly stimulated vesicular transport of brain-derived neurotrophic factor in neuronal cell lines and compensates for the transport deficit in Huntington’s disease models [37]. Similarly, a recent study found HDAC6 null mutation rescued tau-induced microtubule defects in *drosophila* through increased tubulin acetylation [38]. In fact, HDAC6 inhibition alleviates cognitive deficits in transgenic mouse models of AD [39, 40] and also improves memory in a mouse model of tau deposits [41].

Another interesting finding of this study is the better preserved number of processes decorated by polyglutamylated tubulin recognized by the B3 polyglutamylated tubulin antibody which demonstrated significantly increased ratio in the remaining processes positive for α-tubulin. Tubulin polyglutamylation is abundant in neurons which involves the addition of one to six glutamyl units to γ-carboxyl group of glutamate at the C-terminal tail domain of both α- and β-tubulin [42–44]. Because we focused on modifications to α-tubulin, we chose to use the B3 monoclonal polyglutamylation antibody which preferentially recognizes polyglutamylated α-tubulin [45]. However, since this antibody recognizes only polyglutamylated α-tubulin containing side chains with ≥2 glutamate residues [45, 46], it must be emphasized that it does not provide information of all forms glutamylated α-tubulin due to the obvious lack of detection for monoglutamylated form. The function of tubulin polyglutamylation remains poorly characterized partly due to the complex tubulin polyglutamylation patterns [47], but it is believed that tubulin glutamylation is involved in fine-tuning a range of microtubule functions by regulating the binding to microtubule of various microtubule-associated proteins including tau, MAP1A, 1B and 2 and motor proteins including both kinesins and dyneins [48–51]. For example, kinesin-1 motility is increased by tubulin polyglutamylation [52] and *in vivo* study suggested polyglutamylation of α-tubulin as a molecular traffic sign for correct targeting of KIF1 kinesin required for continuous synaptic transmission [51]. Therefore, such an increased ratio of polyglutamylated tubulin in the remaining tubulin-positive processes may help to preserve essential functions of microtubules such as axonal transport. The recent finding of tubulin polyglutamylation stimulated spastin-mediated microtubule severing suggest that tubulin polyglutamylation could act as a signal to control microtubule mass and stability within a cell [53]. However such a signal is likely context specific and the outcomes are mediated by spatially restricted tubulin interactors of diverse nature within the same cell since another study demonstrated that hyperelongation of glutamyl side chains stabilized cytoplasmic microtubules and destabilized axonemal microtubules [54]. It is possible that the increased polyglutamylated tubulin in the soma along with its reduction in the neuronal process observed in human AD brain may represent an adaptation process helping to stabilize the microtubule structures so as to compensate for the overall loss of microtubules.

Comparing the length of tubulin-positive axons in neurons with or without NFTs in AD and control brain revealed that NFT-free neurons demonstrated similar length between AD and control, suggesting there is no specific effects of disease state. We found that NFT formation caused reduced length of axonal processes decorated by α-tubulin and its modified forms in NFT-bearing neurons in both AD and control patients, indicating a specific detrimental and cell autonomous effect of tau pathology on microtubule. This is likely a gradual and chronic process because significant numbers of NFT-bearing neurons still display long axons similar to that of NFT-free neurons. Prior studies demonstrated a selective loss of acetylated tubulin in the NFT-bearing neurons [14]. We did not find such a pattern. Many NFT-bearing neurons with long axons demonstrated similar levels of acetylated tubulin comparable to neighboring NFT-free neurons, while in those NFT-bearing neurons without long axons, acetylated tubulin was detected in the cell body. Similar observations were made for polyglutamylated tubulin as well. These data suggest that the detrimental effects of tau pathology on microtubule are unlikely mediated through the selective reduction of specific posttranslational modifications of tubulin.

## Abbreviations

AD: Alzheimer disease
GAPDH: Glyceraldehyde 3-phosphate dehydrogenase
HDAC6: Histone deacetylase 6
NFTs: Neurofibrillary tangles
